# French-Canadian translation of a self-report questionnaire to monitor opioid therapy for chronic pain: The Opioid Compliance Checklist (OCC-FC)

**DOI:** 10.1080/24740527.2020.1724777

**Published:** 2020-03-02

**Authors:** Clarice Poirier, Marc O. Martel, Mélanie Bérubé, Aline Boulanger, Céline Gélinas, Line Guénette, Anaïs Lacasse, David Lussier, Yannick Tousignant-Laflamme, M. Gabrielle Pagé

**Affiliations:** a Centre de recherche, Centre hospitalier de l’Université de Montréal (CRCHUM), Montreal, Quebec, Canada; b Faculty of Dentistry & Department of Anesthesiology, McGill University, Montreal, Quebec, Canada; c Faculty of Nursing, Université Laval, Québec City, Quebec, Canada; d Centre de recherche du Centre hospitalier universitaire de Québec, Population Health and Optimal Health Practices Research Unit, Québec City, Quebec, Canada; e Department of Anesthesiology and Pain Medicine, Faculty of Medicine, Université de Montréal, Montreal, Quebec, Canada; f Pain Clinic, Centre hospitalier de l’Université de Montréal (CHUM), Montreal, Quebec, Canada; g Ingram School of Nursing, McGill University, Montreal, Quebec, Canada; h Faculty of Pharmacy, Laval University, Quebec City, Quebec, Canada; i Department of Health Sciences, Université du Québec en Abitibi-Témiscamingue, Rouyn-Noranda, Quebec, Canada; j Centre de recherche, l’Institut universitaire de gériatrie de Montréal du CIUSSS du Centre-Sud-de-l’Ile-de Montréal, Montreal, Quebec, Canada; k Département de médecine, Faculté de médecine, Université de Montréal, Montreal, Quebec, Canada; l School of Rehabilitation, Faculty of Medicine and Health Sciences, Université de Sherbrooke, Sherbrooke, Quebec, Canada

**Keywords:** opioids, OCC, French translation, questionnaire, OCC-FC

## Abstract

**Context**: Chronic noncancer pain (CNCP) is a frequent condition among Canadians. The psychosocial and economic costs of CNCP for individuals, their families, and society are substantial. Though opioid therapy is often used to manage CNCP, it is also associated with risks of misuse. The Opioid Compliance Checklist (OCC) was developed to monitor opioid misuse in patients taking opioids for CNCP. The objective of the present study was to provide a French-Canadian translation of the eight-item OCC, the OCC-FC.

**Methods**: The eight-item OCC was translated for use in Québec using published guidelines for the translation and adaptation of self-report measures, including an expert committee and a double forward–backward translation process. A pretest of the adapted eight-item OCC was also conducted among 30 patients with CNCP.

**Results**: A French-Canadian version of the OCC was generated. When ambiguity in the items was detected during expert committee consultation or pretest administration, modifications made were kept to a strict minimum to facilitate future comparisons across studies using the original English and translated French-Canadian version.

**Discussion**: This study provides a culturally adapted tool that will contribute to identifying French-Canadian patients with CNCP who misuse opioids over the course of opioid therapy. This translation of the OCC has the strong potential to be useful in research and clinical settings.

## RÉSUMÉ

**Contexte**: La douleur chronique non cancéreuse est une affection fréquente chez les Canadiens. Les coûts psychosociaux et économiques de la douleur chronique non cancéreuse pour les individus, leurs familles et la société sont considérables. Si le traitement opioïde est souvent utilisé pour la prise en charge de la douleur chronique non cancéreuse, il est également associé à des risques de mauvais usage. La liste de vérification de l'observance du traitement opioïde (OCC) a été élaborée pour surveiller le mauvais usage des opioïdes chez les patients prenant des opioïdes pour la douleur chronique non cancéreuse. L'objectif de la présente étude était de fournir une traduction canadienne-française de l’OCC en 8 points, le OCC-FC.

**Méthodes**: L’OCC en 8 points a été traduit pour être utilisé au Québec selon les lignes directrices publiées pour la traduction et l'adaptation des mesures autorapportées, y compris un comité d'experts et un double processus de traduction - rétrotraduction. Un prétest de l’OCC en 8 points adapté a également été réalisé auprès de trente patients atteints de douleur chronique non cancéreuse.

**Résultats**: Une version canadienne française de l’OCC a été produite. Lorsqu'une ambiguïté dans les énoncés a été détectée par 'lors de la consultation avec le comité d'experts ou de l'administration du prétest, les modifications apportées ont été maintenues au strict minimum afin de faciliter les comparaisons futures entre les études utilisant la version originale anglaise et la version canadienne-française traduite.

**Discussion**: Cette étude fournit un outil culturellement adapté qui contribuera à répertorier les patients canadiens français atteints de douleur chronique non cancéreuse qui font un mauvais usage des opioïdes au cours de leur traitement. Cette traduction de l’OCC présente un grand potentiel d’utilité dans la recherche et les milieux cliniques.

## Introduction

Chronic pain is defined as persistent or recurrent pain lasting longer than 3 months.^1^ The first report produced by the Canadian Pain Task Force was published in June 2019 and provided a snapshot of the prevalence, impact, and management of chronic pain in Canada.^2^ Though this report lists existing successful pain strategies that have been implemented across the country, it also highlights the need for better care and treatment for the many Canadians living with chronic noncancer pain (CNCP).^2^ Data obtained from national surveys conducted between 1994 and 2008 revealed that one in five adults older than age 18 reported living with CNCP.^3^

Among the tools in the therapeutic arsenal of CNCP management is opioid therapy.^4^ Long-term opioid therapy (i.e., opioid use lasting longer than 90 days) is increasingly used in the treatment of CNCP in First World countries,^5^ and in Quebec, long-acting opioids are increasingly prescribed, whereas prescriptions of short-acting opioids are on the decline.^6^ In 2015, a systematic review by Chou and colleagues pointed out the increased risks for overdose, fractures, and myocardial infarction associated with opioid therapy for CNCP.^7^ Most recently, a systematic review conducted by Bialas and colleagues showed that long-term opioid therapy may be beneficial for carefully selected patients presenting with chronic low back pain, diabetic polyneuropathy, and osteoarthritis pain.^8^ Nevertheless, the substantial benefits of long-term opioid therapy for CNCP have been associated with potential risks, including misuse (i.e., the use of opioids contrary to the directed or prescribed pattern of use), with rates averaging between 21% and 29%.^9^ Given this fine balance between optimal pain relief and minimization of opioid-related harms, access to validated tools is critical to monitoring opioid misuse among patients with CNCP over the course of long-term opioid therapy.

A number of tools have been developed and validated to monitor patients on opioid therapy. Some of these tools were designed to monitor adverse effects of opioid medications (e.g., constipation, nausea) or aberrant opioid-related behaviors. *Aberrant behaviors* refers to a wide range of erratic behaviors observed in patients in relation to their prescribed medications, such as aggressively communicating the need for opioid medication, asking for specific opioid medications by name, or frequently calling the clinic or pharmacy regarding opioid prescriptions.^10,11^ Although clinically relevant, it is not always clear whether these aberrant opioid-related behaviors arise from a patient’s misunderstanding of the prescription or from issues related to misuse, addiction, or diversion. Given the potentially deleterious harms and consequences associated with opioid misuse in the context of long-term opioid therapy, self-report tools (i.e., questionnaires) have been developed to assess and monitor opioid misuse over the course of long-term opioid therapy among patients with chronic pain. These tools include the Current Opioid Misuse Measure,^12^ the Pain Medication Questionnaire,^13^ and the Prescription Drug Use Questionnaire, patient version.^14^ Although these tools have proved useful, they are limited due to either their lack of feasibility (e.g., long completion time) or methodological bias (e.g., no or poor content validation and/or information on tool development).

The Opioid Compliance Checklist (OCC) was developed in 2014 by Jamison and colleagues in response to calls for a brief assessment tool designed to assess commonly observed opioid misuse behaviors among patients with CNCP on long-term opioid therapy.^15^ The OCC was also designed to reflect items typically included on an opioid treatment agreement. The questionnaire is intended to be completed by patients and used by prescribing physicians and health care providers.^15^ The original 12-item OCC as well as shorter versions of the questionnaire were validated in large-scale studies across primary- and tertiary-care settings with patients presenting with CNCP.^15,16^ The instrument was designed for American English-speaking populations. To date, a linguistically or culturally adapted version of the OCC is not available to French-Canadian populations. Considering the proportion of Canadians reporting French as their mother tongue (21.4% in 2016), the primary purpose of this study was to develop a French-Canadian translation of the OCC, the OCC-FC.^17^

## Methods

### The Opioid Compliance Checklist

The items of the original OCC were selected following a consensus of experts based on a review of the literature identifying the main components of an opioid therapy agreement.^15^ Though the initial version of the OCC included 12 items, additional validation studies led to the development of a shorter eight-item version in which four items were omitted for their lack of clinical utility in improving predictive power to establish opioid misuse.^16^

All of the OCC items were designed to reflect a “yes” or “no” response over the past month for behaviors associated with misuse of prescribed opioid therapy.^15,16^ As reported in Table 1, which presents the psychometric properties of the self-reported questionnaire, one positive response (yes) to any OCC item was found to predict the likelihood of opioid misuse with a sensitivity of 0.597, a specificity of 0.653, a positive predictive value of 0.381, and a negative predictive power of 0.819.^16^ The area under the curve for all eight items of the OCC with one yes response was found to be 0.645.^16^ The original eight OCC items are listed in Table 2.

**Table 1.** Psychometric properties of the eight-item OCC with a cutoff value of one “yes” response

Area under the curve	0.645
95% Confidence interval	0.562–0.721
*P* value	<0.01
Sensitivity	0.597
Specificity	0.653
Positive predictive value	0.381
Negative predictive power	0.819

Source: Jamison et al.^16^

**Table 2.** Overview of the results of the pretest and changes made to produce the OCC-FC

Items of the original OCC		Penultimate French-Canadian version	Comments from participants who reported that the items should be improved or who did not understand the question	OCC-FC
Title	Opioid Compliance Checklist	Liste de vérification de l’observance aux opioïdes	The word *observance* is not clear (*n* = 29) Given that it refers to the title of the questionnaire, and not a specific item of the checklist, the word observance was kept	Questionnaire de vérification de l’observance aux opioïdes
Definition	N/A	Notez que le terme « médicaments opioïdes » fait référence aux médicaments aussi connus sous le nom d’analgésiques opioïdes, d’opiacés ou de narcotiques que vous prenez pour soulager la douleur (e.g., codéine, fentanyl, hydromorphone, mépéridine, methadone, morphine, oxycodone, tramadol, tapentadol)	Participants are unsure whether they are taking opioids and only know the brand name of their medication. Therefore, they are unable to identify their opioid medication from the list (*n* = 2)	Notez que le terme « médicaments opioïdes » fait référence aux médicaments aussi connus sous le nom d’analgésiques opioïdes, d’opiacés ou de narcotiques que vous prenez pour soulager la douleur. Ces médicaments incluent entre autres la buprénorphine (e.g., Butrans, Suboxone), codéine (e.g., Empracet, Tylenol 3), fentanyl (Duragésic), hydrocodone (e.g., Tussionex, Hycodan), hydromorphone (e.g., Dilaudid, HydromorphContin), mépéridine (Démérol), méthadone (e.g., Metadol, Methadose), morphine (e.g., Statex, MS Contin, Doloral, M-Eslon, Kadian, M.O.S., MS IR), oxycodone (e.g., Oxycocet, OxyNEO, Oxy IR, Targin, Supeudol), tapentadol (Nucynta), tramadol (e.g., Durela, Ralivia, Tramacet, Tridural, Ultram, Zytram)
Possible answers	Yes	Oui	N/A	Oui
	No	Non	N/A	Non
Instructions	Over the past month, have you:	Au cours du dernier mois, avez-vous:	The instructions should be repeated before each of the eight items (*n* = 2)	Au cours du dernier mois, avez-vous:
1	Taken your opioid medication other than the way it was prescribed?	Pris vos médicaments opioïdes autrement que prescrits?	It is unclear whether this item assesses whether patients are buying their medication “on the streets” or using someone else’s (*n = *10). The question should be more specific (i.e., it should ask directly whether patients are using the right dose at the right time; *n* = 4)	Pris vos médicaments opioïdes différemment de la façon dont ils vous ont été prescrits?
2	Used more than one pharmacy to fill your opioid prescriptions?	Utilisé les services de plus d’une pharmacie pour faire remplir vos prescriptions d’opioïdes?	“Faire remplir une prescription”, c’est le médecin (*n* = 1) Answered “yes” to this item but explained that they go to one pharmacy (*n* = 1)	Utilisé les services de plus d’une pharmacie pour faire remplir vos ordonnances d’opioïdes?
3	Received opioid prescriptions from more than one provider?	Reçu des prescriptions d’opioïdes de plus d’un professionnel de la santé?	This item needs clarification (*n* = 1). “Professionnel de la santé” refers only to the doctor at the pain clinic (*n* = 1)	Reçu des ordonnances d’opioïdes de plus d’un professionnel de la santé?
4	Lost or misplaced your opioid medications?	Perdu ou égaré vos médicaments opioïdes?	The wording is strange (*n* = 2)	Perdu ou égaré vos médicaments opioïdes?
5	Run out of your pain medication early?	Manqué de médicaments contre la douleur plus tôt que prévu?	This item is very similar to the first one (*n* = 1). This item assesses whether patients are “proactive” in their treatment (*n* = 1)	Manqué de médicaments contre la douleur plus tôt que prévu?
6	Missed any scheduled medical appointments?	Manqué des rendez-vous médicaux?	“Rendez-vous médicaux” only involves my appointment at the pain clinic (*n* = 3). It is unclear whether “rendez-vous médicaux” implies appointment with a physiotherapist (*n = *1) or an occupational therapist (*n* = 1)	Manqué des rendez-vous médicaux?
7	Borrowed medication from others?	Emprunté des médicaments opioïdes à d’autres personnes?	N/A	Emprunté des médicaments opioïdes à d’autres personnes?
8	Used any illegal or unauthorized substances?	Utilisé toute substance illégale ou non-autorisée?	This item includes the use of cannabis (i.e., cannabis is considered as an illegal or unauthorized substance; *n = *12) It should be specified by whom the substance is authorized or not (for example, the authority, a doctor, workplace regulations; *n* = 2)	Utilisé toute substance illégale ou non autorisée?*
Specifications	N/A	N/A		*Toute substance qui n’est pas permise par la loi. Par exemple, au Canada, le cannabis est légal donc autorisé et ne devrait pas être pris en compte

For items 2 and 3, the term *prescriptions* was changed to *ordonnance* by the expert committee for increased accuracy. The former term refers to a recommendation or therapeutic advice from a professional, whereas the latter term refers to the actual written document on which the prescription is written.

The validity (i.e., the extent to which the instrument measures what it is supposed to measure) of the use of this screening tool was assessed in two large prospective studies.^15,16,18,19^ Patients with CNCP were recruited from a tertiary urban hospital (*N* = 157) for the original validation study and from eight primary care centers (*N* = 253) for the second validation study.^15,16^ Among the criteria used to measure validity are face validity, content validity, and criterion validity. In a systematic review published by Lawrence and colleagues in 2017, the quality of both of these validation studies was assessed as “high” based on the methodology defined in the Scottish Intercollegiate Guidelines Network checklist.^18,20^ Taking less than 2 min to complete, the OCC was described by the same authors as a “promising tool which may offer more functionality for both screening and predicting, being shorter, and having been developed and further validated in good quality studies.”^18^

### Procedures for the Translation of the OCC

Guidelines for the translation and adaptation of self-report measures were published by Beaton and colleagues in 2000.^21^ The French translation of the OCC was accomplished in accordance with these guidelines. Permission to translate and adapt the eight-item version of the OCC was granted by the corresponding author of the questionnaire.

#### Step 1: French Translation

Forward translation represents the first step of the translation process. The completion of this stage was achieved by two independent bilingual individuals speaking Canadian French as their first language. The first translator was a trainee in the field of chronic pain (T1) and the second was a French linguist without prior knowledge of the concepts of interest (T2). A report summarizing the rationale for their choices for translating the items and comments with regards to challenging phrases and uncertainties was produced by each of the translators.

#### Step 2: Forward Translation Synthesis Meeting

The second step included a synthesis of the translations that were made by the two translators in stage 1 (T1 and T2 versions). The two forward translators and the research coordinator met through a web-based screen-sharing system (Zoom.us) to examine the reports produced in the first step. At the end of this meeting, the two translators had reached a consensus and provided a common French-Canadian version of the questionnaire (T-12 version).

#### Step 3: Back-Translation and Back-translation Synthesis Meeting

Two independent bilingual translators from Canada who spoke English as their first language completed this step consisting of a back-translation. The first back-translator was a clinician in the field of chronic pain (BT1) and the other was a certified English linguist without a biomedical background (BT2). Both were blinded to the original version of the instrument. BT1 and BT2 translated the questionnaire back into its original language (English) using the version T1/T2 produced in the second step. As in the first step, each of the back-translators submitted a report detailing their rationale for the translation of each item.

The reports obtained following the back-translation were compared in a meeting held between the two back-translators and the research coordinator (using a web-based screen-sharing system) to highlight unclear wording.^21^

The third step was followed by the elaboration of a global report including the original version of the OCC, the two French-Canadian translations and comments of the translators, the T1/T12 version, and the two English back-translations and comments of the back-translators.

#### Step 4: Expert Committee

The expert committee was composed of researchers in the field of chronic pain and opioids; an epidemiologist; psychologists; nurses; physicians, including a geriatrician and an anesthesiologist; a physiotherapist; a pharmacist; and the aforementioned four translators (*N* = 13). In line with Beaton and colleagues’ guidelines recommending that the developers of the original tool be in close contact with the expert committee, one of the expert committee members (M.O.M.) was also part of the group of researchers who developed the original OCC.^21^

The global report produced in the third step was sent to all participating experts. The expert committee then met using a web-based screen-sharing system with the objective of producing a penultimate French-Canadian version of the OCC that achieved equivalence between the original and the translated questionnaire in four major areas: (1) semantic equivalence (i.e., the meaning of the words), (2) idiomatic equivalence (i.e., the formulation of equivalent items for language-specific expressions), (3) experiential equivalence (i.e., the replacement of items that are seeking to capture an experience of daily life by expressions that are adapted to target the culture), and (4) conceptual equivalence (i.e., the definition of concepts that are common to both cultures).^21^

#### Step 5: Pretest

In accordance with the published guidelines for the translation of self-reported measures, the French-Canadian version of the OCC was pretested in a sample of 30 French-Canadian patients treated in a tertiary-care pain center in the province of Quebec.^21^ The study was approved (no. 19.109-YP) by the Ethics Review Board of the Center hospitalier de l’Université de Montréal. The following inclusion criteria were applied: (1) having pain for a minimum of 3 months, (2) being 18 years of age and older, (3) speaking French as a first language, and (4) being on prescribed opioid therapy for a minimum of 30 days. The 30 days of opioid therapy criterion was based on the OCC instructions according to which opioid-related behavior must be measured over a 1-month time frame.^15^

Between July and September 2019, 30 participants were recruited using a prospective convenience sampling method. Informed consent was obtained from patients who met the inclusion criteria. Face-to-face cognitive interviews were conducted while participants completed the penultimate French-Canadian version of the eight-item OCC. Participants were asked to verbalize their thought process while completing the OCC and were prompted to explicitly describe their understanding of each item in order to ensure that the words and concepts were understood without ambiguity. Examples of specific questions that were asked of the participants included the following: “How would you say this in your own words?”; “What does this word mean to you?”; “Did you find it difficult to answer this question?”; “How would you phrase the question to make it easier for people to understand?”

#### Step 6: Final Versions

After reviewing the results obtained in the pretest, members of the expert committee agreed upon slight modifications (see Table 2) to the French-Canadian version. The final version of the OCC-FC is presented in the Appendix.

## Results

Steps 1 to 4 were conducted during spring and summer of 2019. Recruitment of 30 patients for the fifth step (pretest) took place between July and September 2019. Characteristics of participants who took part in the interviews are presented in Table 3. Participants’ mean age was 56.2 years, 56.7% were men, and all participants were white. Slightly more than half of participants (53.3%) had postsecondary education. In addition, 53.3% of participants reported disability status and 33.3% were retired. The mean duration of pain reported by the participants was 19.3 years and 56.7% reported multiple pain sites. On average, the participants had been on opioid therapy for 10.8 years (range = 0.5–42 years).

**Table 3.** Characteristics of the study participants (*n* = 30)

Characteristics	No. (%)^a^
Age (years), mean ± SD (range)	56.2 ± 13.3 (22–73)
Gender	
Male	17 (56.7)
Female	13 (43.3)
Ethnicity	
Caucasian	30 (100.0)
Employment status	
Short- or long-term disability	16 (53.4)
Retired	10 (33.3)
Other	4 (13.3)
Highest education level attained	
Elementary or high school	14 (46.7)
College, CEGEP, or other nonuniversity certificate diploma	10 (33.3)
University certificate, bachelor’s degree, or diploma above bachelor’s level	6 (20.0)
Duration of pain (years), mean ± SD (range)	19.33 ± 13.20 (3–46)
Average pain intensity in the past 7 days (0–10), mean ± SD (range)	6.84 ± 1.98 (3–10)
Duration of opioid therapy (years), mean ± SD (range)	10.8 ± 8.4 (0.5–42)

^a^Unless otherwise specified.

An overview of the results of the pretest and the modifications made to obtain the final French-Canadian version are presented in Table 2. First, a definition of the term *opioid medication* accompanied by a list of generic medication names available in Canada was added to the pre-final version by the expert committee (step 4). Some participants were unfamiliar with the term opioids, and it was difficult for them to recall the names of their medications. However, most were able to recognize the name of their medication from the list provided in the questionnaire. Examples of brand names were also added to the final French-Canadian version (step 6) following the pretest (step 5) because two participants were unable to recognize the generic name of their opioid medication.

Though most items of the penultimate version of the questionnaire were clear and well understood by the participants, two items stood out as needing more explanation to be understood by participants. The first item of the penultimate version (Taken your opioid medication other than the way it was prescribed) was misinterpreted by the respondents. Though this item was intended to explore whether participants had been using their medication other than the way it had been prescribed (e.g., using more opioids than prescribed, using opioids for reasons other than pain, or changing the route of administration), some participants thought that the question was about their use of illegal drugs. The wording in the final version was modified to better represent the intent of the question. The last item probing participants on their use of illegal drugs was also modified to clarify the legal status of cannabis given its recent (October 2018) legalization in Canada.^22^ Finally, the term used to refer to opioid prescriptions (items 2 and 3) was changed in step 6 in favor of a correct term instead of a more accessible but incorrect French term (see footnote Table 2).

Results of the pretest showed that the questionnaire was globally understood by participants and did not pose particular challenges that needed to be addressed. Only minor modifications were made to few items for clarification purposes.

## Discussion

This study describes the various steps that were taken to develop a French-Canadian version of the OCC (OCC-FC). It is important to have easy-to-administer, sensitive, self-report tools to detect the risk of opioid misuse, particularly among patients with chronic pain. As described earlier, opioid therapy is not without risks; one of those risks is the development of opioid misuse or dependence. However, in the context of the opioid crisis, there is increasing reticence to prescribe opioids to patients with chronic pain who could greatly benefit from this therapeutic approach. Indeed, attention has recently been drawn to the negative consequences for patients with chronic pain of too-restrictive opioid prescription practices.^23,24^ Being able to monitor one’s response to opioid therapy, including the presence of misuse behaviors, is essential.

As previously described, results obtained through cognitive interviewing (step 5, pretest) failed to reveal any major difficulty with regards to the penultimate French-Canadian version of the OCC generated by the expert committee (steps 4–5). Hence, the modifications made to obtain the final version were minor and related to two items. The absence of any meaningful differences between the original English and French-Canadian versions will facilitate comparisons across studies.

The sample used for cognitive interviews was diverse in terms of gender, education level, age, and pain duration. The clarity of the items did not seem to vary according to sociodemographic characteristics measured.

Though our inclusion criteria regarding the duration of pain (i.e., having chronic pain for a minimum of 3 months) correspond to the International Association for the Study of Pain’s definition of chronic pain, they differ from the inclusion criteria used in both validation studies of the OCC (i.e., “had pain for >6 months’ duration”).^15,16^ Again, this distinction had no impact because the shortest duration of pain in our sample was 3 years. Finally, the characteristics of our sample resemble those from both samples used in the validation studies of the OCC in terms of age, gender, pain sites, and average pain intensity.^15,16^

### Limitations

Though strict guidelines for the translation of self-reported measures were followed, a number of study limitations must be acknowledged. First, the psychometric properties of the OCC-FC have yet to be assessed. Not only will validation studies using the OCC-FC permit assessment of its validity, sensitivity, and specificity, they will also enable its comparison with the original version. Second, all 30 participants in the present study were white. This lack of ethnic diversity within the present sample can be explained by one of the inclusion criteria, which specified that participants must speak French as a first language. Efforts should be made to include French-speaking individuals from various ethnic backgrounds and other Canadian provinces in future studies testing the OCC-FC. In addition, a range of education levels was represented in our sample, but we did not directly measure health literacy. Third, all data were obtained from patients at a single pain management clinic in a tertiary urban hospital. Multicenter validation studies involving patients with CNCP followed in other settings, such as primary and secondary care, would allow testing the OCC-FC among a more diverse population. Fourth, the OCC has some limitations.^15,16^ Among the limitations inherent to the original questionnaire are its relatively low sensitivity, specificity, and positive predictive value, which might result in clinicians refraining from using this tool.^16^ Finally, as all self-report measures, the scores obtained using the OCC depend on patients answering truthfully.^16^ Hence, the OCC should be considered along with other tools to assess opioid misuse.

## Conclusion

Despite some of the limitations noted above, the OCC represents a promising self-report questionnaire that could be useful for the screening and/or monitoring of opioid misuse among patients with CNCP on long-term opioid therapy.^15,16,18^ This study provided a translated tool that will be useful for the assessment of opioid misuse among French-Canadian patients with CNCP prescribed opioid therapy, in both research and clinical settings. It is a self-report tool that can be quickly administered in clinical settings. Our next steps will include the former validation of the translated tool in various populations and across different settings to examine the psychometric properties of this tool. It is thus timely to publish this translation at this time so that other research teams can conduct validation studies in parallel using different populations across Canada.
